# Comparison of day surgery between varicose veins with and without superficial venous thrombosis below knee: a propensity score-matched analysis

**DOI:** 10.1186/s12872-023-03398-2

**Published:** 2023-08-03

**Authors:** Jiatang Xu, Xiaolin Xu, Jing Tian, Minyi Huang, Zuqi Xia, Xianghui Luo, Junmeng Zheng, Kai Huang

**Affiliations:** 1 Department of Cardiovascular Surgery, Sun Yat-Sen Memorial Hospital, Sun Yat-Sen University, Haizhu District, No.33, Yingfeng Road, Guangzhou, 510000 Guangdong Province China; 2 Zhongshan School of Medicine, Sun Yat-Sen University, No.58, Zhongshan Rd.2, Guangzhou, 510080 Guangdong Province China; 3 Department of Ultrasound, Sun Yat-Sen Memorial Hospital, Sun Yat-Sen University, Haizhu District, No.33, Yingfeng Road, Guangzhou, 510000 Guangdong Province China; 4 Operating Theatre, Sun Yat-Sen University, No.58, Zhongshan Rd.2, Guangzhou, 510080 Guangdong Province China

**Keywords:** Varicose veins, Day surgery, Superficial venous thrombosis, Propensity score matching

## Abstract

**Objectives:**

Development of endovenous treatment and sclerotherapy technology makes it feasible for clinicians to treat varicose veins (VV) through day surgery (DS). Superficial venous thrombosis (SVT) of lower extremities is a common complication of VV. This study aimed to investigate whether the existence of SVT below knee affect the safety and efficacy of DS for VV patients.

**Methods:**

This is a single-center retrospective study. Clinical data of 593 VV patients was retrospectively analyzed. Raw data were matched by the using of propensity score matching model. Operation time, technical failure, postoperative DVT, skin burns, saphenous nerve injury, subcutaneous induration, and bleeding were compared between the groups. Also, we compared VV recurrence, SVT formation, DVT events and the change of VCSS score with 12 months.

**Results:**

Fifty-nine patients complicated with SVT below knee were matched with 118 patients had VV only. Perioperative and follow-up outcomes were similar in both groups except for the number of incisions (median = 6 [5, 7] VS median = 4 [4, 5], *P <* 0.001). Both groups experienced a great decrease in VCSS score.

**Conclusion:**

We systematically compared the clinical outcomes of DS in VV patients. Our results indicate DS is safe and effective for patients with VV, whether accompanied by SVT below the knee.

**Trial registration:**

The ClinicalTrials.gov identifier for this trial is NCT05380895 (retrospectively registered).

**Supplementary Information:**

The online version contains supplementary material available at 10.1186/s12872-023-03398-2.

## Background

Varicose veins (VV) of lower extremities are considered as common diseases in outpatient and inpatients. Nearly 25% of the global adult population suffer from VV, of which, the prevalence of VV in China reaches 8.9%, affecting more than 100 million people [1, 2]. Based on this, varicose veins emerge as a highly prevalent condition and a significant public health concern worldwide, imposing substantial economic and medical burdens on both individuals and healthcare systems.

Compared to inpatient surgery (IS), which previously used for treating VV, day surgery (DS) offers higher medical efficacy and lower healthcare costs [3, 4]. Moreover, advancements in endovenous technology have made it possible for clinicians to effectively treat VV through DS [2, 5, 6]. DS for VV patients presents a viable solution to mitigate the inherent contradiction between the growing medical demand and the scarcity of medical resources [7, 8].

Superficial venous thrombosis (SVT) is a frequent complication of VV. It manifests as pain, erythema, and typically a palpable nodular mass. Although SVT can occur independently, VV remains the primary risk factor. Previous studies have indicated that the likelihood of SVT in VV patients ranges from 4 to 59%, and up to 80% of SVT cases are accompanied by VV [9–11]. For a long time, SVT has been regarded as a benign, self-limiting condition, often receiving insufficient attention in clinical decision-making. However, recent research indicates a strong association between SVT and the development of venous thromboembolism. In particular, when SVT involves the blood vessels that connect to the deep venous system [10, 12, 13]. The latest guidelines from the journal CHEST recommend a 45-day course of anticoagulant therapy for SVT patients to reduce the risk of VTE progression. Additionally, surgical interventions are also employed as treatment options for SVT [10, 14, 15].

Most scholars believe that VV patients combined with SVT require a period of anticoagulant therapy before surgical treatment can be performed; however, the evidence supporting this viewpoint is not of high grade (Class IIa, grade C) [16]. Moreover, with the development of minimally invasive technology and DS, this view may face huge challenges.

SVT patients typically require a period of standardized anticoagulant therapy, while DS is currently considered the mainstream treatment for VV. However, it remains unclear whether the presence of SVT affects the safety and efficacy of DS for VV patients, which may lead a change in treatment strategy and the implementation of anticoagulant treatment for SVT prior to DS. To date, there is no research on this issue.

It remains unclear the impact of SVT on the efficacy of DS for varicose veins patients. To comprehensively evaluate the feasibility and efficacy of DS for VV patients complicated with SVT, we conducted this study and systematically compared it to patients with VV only. Considering particles of thrombus of thigh can be propagated into the deep venous system by pushing the device forward through the affected vein, only patients with SVT below knee were included in our study.

## Method

### Patients

This is a single center retrospective cohort study, which retrospectively analyzed the clinical data of 593 VV patients treated in Sun Yat-sen Memorial Hospital of Sun Yat-sen University from 2015 to 2021. Doppler ultrasound, combined with clinical examination, was used in the diagnosis of SVT. By consulting the electronic medical record, the demographic and clinical information such as patient gender, age, weight and basic diseases were extracted. Preoperative evaluation of VV patients included CEAP grade and VCSS score [17, 18]. To reduce the possible selection bias, a propensity score matching model (PSM) was used to balance the baseline data of the two groups of patients [19]. Patients complicated with SVT (59) were matched at a ratio of 1:2 with patients suffered VV only (118) finally.

Inclusion criteria: VV patients with the CEAP grade of C2-C5 and at the age 18 to 75; SVT limited to calf.

Exclusion criteria: (1) Patient has a history of Deep vein thrombosis (DVT) or is suffering from DVT; (2) Patients with liver and renal failure; (3) Patients in hypercoagulable state, such as patients with the history of tumor, in pregnancy or had a recent major surgery; (4) Patients with anticoagulant therapy before operation; (5) Patients with active ulcer; (6) Patients allergic to foam sclerosant.

We conducted this study in line with the STROBE guidelines. All methods were carried out in accordance with the declaration of Helsinki. Since it is hard to obtain the subject's informed consent, and the research project does not involve personal privacy or commercial interests, written inform consent was waived by Ethics Committee of Sun Yat-sen Memorial Hospital. This study has been approved by ethics committee of Sun Yat-sen Memorial Hospital. The ClinicalTrials.gov identifier for this trial is NCT05380895.

### Day surgery procedure

1. Examined the shape of great saphenous vein (GSV) by the using of Doppler ultrasound (PHILIPS, Holland) to clear whether it was accompanied with SVT, then mark the site of SVT.
2. Under the guidance of Doppler ultrasound, punctured the medial aspect of the knee's GSV and inserted a 6F vascular sheath (COOK, USA).
3. Inserted a microwave ablation catheter (Nanjing ECO, Nanjing, China) through the vascular sheath, positioning it at least 2 cm away from the junction of the GSV and deep vein.
4. Injected swelling anesthetic solution (500 ml of normal saline mixed with 25 ml of 2% lidocaine and 20 units of adrenaline) into the tissue around the GSV under the monitoring of Doppler ultrasound.
5. Performed intermittent microwave ablation on the trunk of GSV using a microwave therapy apparatus (Nanjing ECO, Nanjing, China) with 65W energy.
6. Injected Polidocanol Injection (Hameln pharmaceuticals GmbH, Hameln, prepared in term of Tessari method) into the site of marked lesion.
7. Made a 0.5 cm incision to hook out the superficial varicose veins of calf, combining with ligation and stripping.
8. Made a 0.5 cm incision on the skin at the site of SVT, hook out the superficial varicose veins, harvest the thrombus, combined with ligation and stripping.
9. Set pressure dressing on the lower limb by elastic bandage.

All patients were suggested to wear graduated compression stockings for the next one month. For patients with suffered pain, 0.1 g celecoxib would be taken every 12 h after discharge for 3 days. For patients complicated with SVT, rivaroxaban 10 mg QD was taken orally for one week after discharge.

### Clinical outcomes

Perioperative clinical outcomes included operation time, number of incisions and perioperative adverse events. Perioperative adverse events included postoperative DVT, technical failure, skin burns, saphenous nerve injury, subcutaneous induration, and bleeding requiring intervention. Technical failure is defined as failure of venipuncture or failure to complete the operation after venipuncture.

Follow-up outcomes included VV recurrence, SVT formation, and DVT events. Also, we executed VCSS scores on patients at the third, sixth and twelfth month after operation. VCSS score was calculated in the term of 10 items. The higher the score is, the worse the lower limb veins are.

### Follow‑up

Follow-up assessments were conducted at the third, sixth, and twelfth month after operation, during which the VCSS socre was obtained to evaluate the postoperative condition of the patients. Doppler ultrasound was used to objectively evaluate the follow-up outcomes, included VV recurrence, SVT formation, and DVT events. For most patients, follow-up was carried with their regular outpatient appointments, and the results were recorded in their outpatient medical records. And for those were not able to return for follow-up, such as those residing too far away from our institution, we conducted phone calls with well-trained physicians to collect the necessary information. We carried the last follow-up in December 2021.

### Statistical analysis

To minimize selection bias and ensure comparability of baseline data between the two groups, we performed PSM. The propensity scores of each patient were calculated using a logistic regression model, and a 1:2 matching ratio was applied to match the propensity scores of the two groups. The value of caliper was 0.1. The baseline variables matched in our model included gender, age, weight, combined hypertension, CEAP grade and preoperative VCSS score.χ2 test or Fisher’s exact test were used to compare categorical variables, student’s t test or Wilcoxon rank sum test were used to compare continuous variables. Binary outcomes were calculated by logistic regression. All statistical tests were two tailed, *P <* 0.05 was considered statistically significant. Our statistical analyses were carried out by the using of STATA 15 (STATACrop).

### Patient and public involvement

By the reason of the retrospective and observational nature of the study, the development of the research question and outcome measures have not been informed by patients. Similarly, patients are not involved in the conduct to and recruitment of the study.

## Results

### Patient characteristics and propensity score matching

**Fig. 1:**
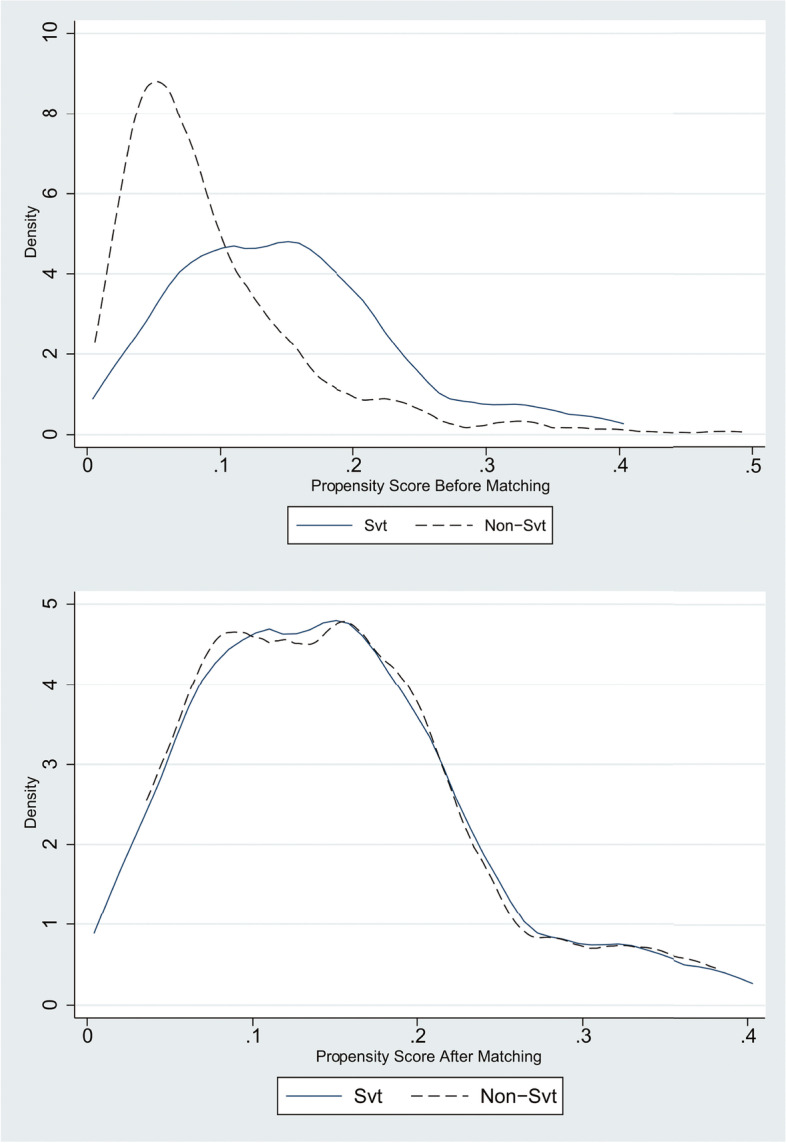
Distribution of propensity score before and after matching

Five hundred ninety-three patients diagnosed with VV were treated by DS, among which 59 patients had the additional complication of SVT. Raw data were analyzed by the using of PSM with a caliper set to 0.1. Matching results are summarized in Fig. 1. Patients complicated with SVT (59) were matched at a ratio of 1:2 with patients who had VV only (118). The baseline characteristics of participant patients before and after PSM were depicted in Table 1. Following PSM, the two groups exhibited similar baseline clinical characteristics and distribution of propensity scores.

**Table 1:** Patients’ baseline characteristics before and after PSM

Characteristics	Before matching			After matching		
*N*	Non-SVT	SVT	*p*	Non-SVT	SVT	*p*
	534	59		118	59	
Age, mean ± SD	55.96 ± 13.18	60.16 ± 9.61	0.003	59.58 ± 10.42	60.16 ± 9.61	0.715
Sex, male, (%)	183(34.26)	22 (37.28)	0.608	43 (36.44)	22 (37.29)	0.912
Weight, [IQR]	62.75 (56.00–71.50)	61.50 (55.50–70.00)	0.764	63.00 (58.00–72.00)	61.50 (55.50–70.00)	0.572
Diabetes, (%)	30(5.62)	2(3.39)	0.678	5(4.24)	2(3.39)	0.785
Hypertension, (%)	85 (15.92)	9(15.25)	0.895	19 (16.10)	9 (15.25)	0.884
VCSS, mean ± SD	6.50 ± 1.56	7.22 ± 1.50	< 0.01	7.06 ± 1.36	7.22 ± 1.50	0.475
CEAP grade, N, (%)			< 0.01			0.470
C2	22 (4.12)	0 (0)		0 (0)	0 (0)	
C3	144 (26.97)	0 (0)		11 (9.32)	0 (0)	
C4a	197 (36.89)	21 (35.59)		32 (27.12)	21 (35.59)	
C4b	115 (21.54)	30 (50.85)		40 (33.90)	30(50.85)	
C5	56 (9.93)	8 (13.56)		35 (29.66)	8 (13.56)	

Continuous variables that do not conform to normal distribution were represented by median and interquartile range, continuous variables that conform to a normal distribution were represented by mean and standard deviation representations; Categorical variables were expressed by quantity and percentage

### Perioperative outcomes in SVT and non‑SVT patients

The operation time in the SVT group was statistically paralleled to that of the non-SVT group, with no significant difference observed (median = 41 [39,45] VS median = 41 [38,44], *P =* 0.726). Whereas, patients complicated with SVT had more incisions compared to those with VV only (median = 6 [5,7] VS median = 4 [4,5], *P <* 0.001). There was one reported case of technical failure in the non-SVT group, but the difference between the two groups was not significant (*P =* 0.553). In this reported case, the operator attempted to puncture the trunk of GSV but was unsuccessful. The incidence of adverse events was low, with the exception of subcutaneous induration. However, all instances of subcutaneous induration, predominantly along the GSV, were temporary and resolved after a short period of conservative treatment.

Among patients with VV only, perioperative adverse events consisted of 2 cases (1.69%) of burns, 2 cases (1.69%) of saphenous nerve injury, 46 cases (38.98%) of subcutaneous induration, and 1 case (0.85%) of DVT occurrence. And among patients complicated with SVT, perioperative adverse events included 2 (3.39%) of saphenous nerve injury, 25 (42.37%) of subcutaneous induration and 1 (1.69%) of DVT occurrence.

Neither group experienced bleeding that requiring intervention, and the most frequent complication except for subcutaneous induration was saphenous nerve injury with an incidence of 2.26%. There was no significant difference between the two groups in the incidence of perioperative adverse events (Table 2).

**Table 2:** Perioperative outcomes in propensity score matched cohort

Perioperative outcomes	Non-SVT	SVT	*P*
Operation time, median [IQR]	40.00 [38.00–44.00]	41.00 [39.00–45.00]	0.726
Number of incisions, median [IQR]	4 [4, 5]	6 [5–7]	< 0.01
Technical failure, N, (%)	1 (0.85)	0 (0)	1.000
Burns, N, (%)	2 (1.69)	0 (0)	0.553
Intraoperative bleeding, N, (%)	0 (0)	0 (0)	1.000
Saphenous nerve injury, N, (%)	2 (1.69)	2 (3.39)	0.858
Subcutaneous induration, N, (%)	46 (38.98)	25 (42.37)	0.664
DVT occurrence, N, (%)	1 (0.85)	1 (1.69)	1.000

### Long‑term outcomes in SVT and non‑SVT patients

**Fig. 2:**
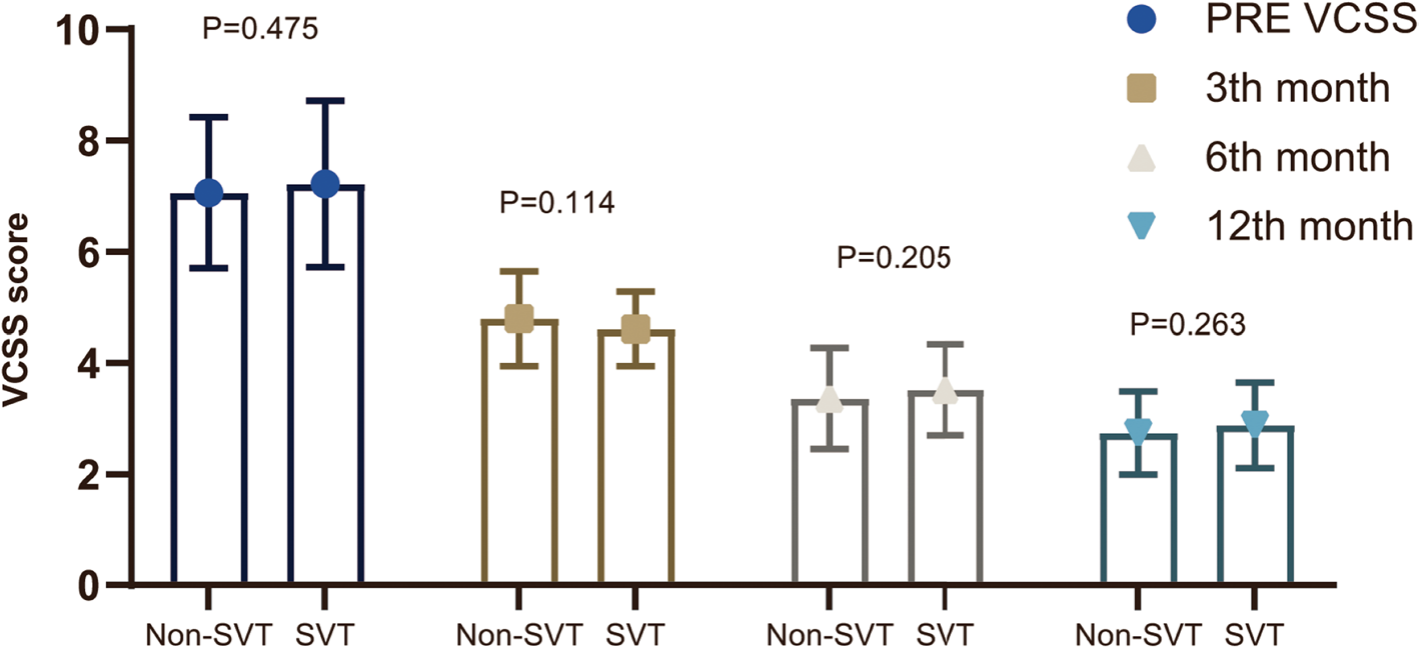
VCSS score during follow-up

Based on the 12-month follow-up, the mean VCSS score for patients with VV only were 4.79, 3.36, and 2.74 at the 3rd, 6th, and 12th month, respectively, which were almost same as patients complicated with SVT (4.61, 3.52 and 2.63), as showed in Fig. 2.

AS showed in Table 3, VV recurrence occurred in 17 patients in group suffered VV only and 7 patients in SVT group during follow-up (14.41% VS 11.86%, *P =* 0.641). No difference was found on the rate of SVT formation between SVT and non-SVT groups (5.08% VS 3.39%, *P =* 0.892). Importantly, no cases of DVT were reported during the follow-up period.

**Table 3:** Long-term outcomes in propensity score matched cohort

Long-term outcomes	Non-SVT	SVT	*P*
Recurrence of VV, N, (%)	17 (14.41)	7 (11.86)	0.641
Formation of SVT, N, (%)	4 (3.39)	3 (5.08)	0.892
DVT occurrence, N, (%)	0 (0)	0 (0)	1.000
VCSS score, 3th month, mean ± SD	4.79 ± 0.85	4.61 ± 0.67	0.114
VCSS score, 6th month, mean ± SD	3.36 ± 0.91	3.52 ± 0.82	0.205
VCSS score, 12th month, mean ± SD	2.74 ± 0.75	2.88 ± 0.77	0.263

## Discussion

With a growing awareness that SVT can lead to DVT or even more serious consequences, the treatment of SVT has been paid more attention though it is a benign disease. Researchers started to explore the anticoagulant therapy of SVT. The journal Chest recommends using a daily dose of 2.5 mg fondaparinux for anticoagulation therapy to treat SVT [14]. Also, rivaroxaban has been shown to be non-inferior to fondaparinux in preventing the combined efficacy endpoint of thrombus progression, SVT recurrence, VTE events, and mortality in a randomized controlled trial involving 406 people [20]. In any case, SVT does seem to be a disease requiring a period of standardized anticoagulant therapy.

From initial traditional surgery to endovascular ablation and foam sclerotherapy, surgery has always been primary treatment approach for varicose veins [2, 21–23]. With the development of endovenous technology, DS has gradually emerged as the mainstream of surgical treatment of varicose veins. DS for varicose veins can ensure both safety and efficacy, and significantly reduce the average medical cost and length of hospital stay compared to IS [5, 24, 25]. Our study aims to explore whether DS remains effective and safe for VV patients complicated with SVT. To the best of our knowledge, this is the first study to analyze the safety and efficacy of DS specifically for VV patients complicated with SVT.

In our study, data of 593 VV patients who underwent DS were retrospectively reviewed. Among them, 59 patients with SVT and 118 patients without SVT were finally matched. Perioperative clinical outcomes and follow-up outcomes were compared between two groups. As far as we can tell, it was the first cohort to explore the impact of SVT on the efficacy of DS for VV. Therefore, the results of our study provide strong evidence regarding the safety and efficacy of DS for VV patients with SVT.

PSM was performed from a large volume of data preexisted to balance the baseline characteristics between SVT and non-SVT group. As a retrospective cohort study, the raw baseline data of the two groups were unevenly distributed in age, VCSS score and CEAP grade. Previous studies have indicated that SVT is more prevalent among the elderly population. And worse VCSS score, as well as higher CEAP grade in SVT group may be attributed to the aggravation of venous symptoms by SVT and the residual pigmentation of local skin after the acute phase [10, 26].

Both SVT and non-SVT patients demonstrated similar clinical characteristics and distribution after matching, which suggested less likelihood of selection bias in studying the outcomes. In our propensity score matched cohort, patients in SVT group had a higher number of surgical incisions compared to patients in non-SVT group. This can be attributed to surgeons tend to make an incision on the surface of SVT in shank for embolectomy for SVT cases. Though embolectomy was performed during surgery, it will not extend operation time, making operation time of two groups comparable. Most incisions were located in shallow skin and less than 2 cm, and these incisions generally healed quickly and resulted in a short recovery time.

Perioperative outcomes indicate the safety and efficacy of DS for VV patients with SVT. The perioperative outcomes were acceptable in both SVT and non-SVT patients. No significant difference in the incidence of technical failure was showed in our research, as well as major perioperative adverse events. The occurrence of major adverse events such as skin burns, saphenous nerve injury, and postoperative deep vein thrombosis (DVT) was low and could be effectively managed with conservative therapy. Additionally, no bleeding requiring intervention occurred, which further suggested the safety of DS for both groups.

In our study, we focused on the high incidence of postoperative subcutaneous induration in both groups (38.98% in the non-SVT and 42.37% in the SVT). Induration along the trunk of GSV in this research, as in previous studies, has a high incidence rate in endovenous ablation, but mostly dissolved within 4 weeks (i.e. at the first review after discharge) [27]. Traditionally, SVT was regarded as a disease with high risk of VTE and required standardized anticoagulant therapy for a period of time. However, our finding suggested that in VV patients with SVT, DS can be safely performed without the need for standardized anticoagulation before the operation.

Moreover, although no significant difference in incidence of postoperative DVT was find in our research between the two groups, two patients still experienced postoperative DVT. By reviewing the surgical records, we speculated that this could be attributed to the excessive use of foam sclerosing agents during the operation [28, 29]. Both patients suffered asymptomatic distal DVT patients, and the thrombus disappeared after a short period of after short-term therapeutic dose anticoagulation.

During the long-term follow-up, both groups showed a great decrease in VCSS score. VV clinical recurrence was observed in 7 (11.86%) patients with SVT and 17 (14.41%) patients without SVT, which suggested satisfactory long-term efficacy. SVT recurrence occurred in 3 (5.08%) patients with SVT and 4 (3.39%) patients without SVT during follow-up, and all cases were cured after treated with topical Hirudoid. Importantly, no patients suffered DVT during follow-up which also demonstrated the long-term safety of DS in VV patients with SVT.

Several limitations in the study should be addressed. First, being a retrospective study, we cannot eliminate all possible biases through PSM. For instance, the majority of patients included in our study had CEAP grades ranging from C2 to C5, while the more serious patients with active ulcer have not been adequately analyzed. Therefore, further research is required to investigate the safety and efficacy of DS for those patients with active ulcer (C6). Second, considering particles of SVT of thigh can be propagated into the deep venous system in endovenous ablation, only patients with SVT below knee were included in our study. Third, not all patients underwent lower extremity ultrasonography during follow-up, which means that there is a possibility that some cases of asymptomatic isolated DVT were not documented. At last, our study is a single center study, and the clinical outcomes observed may not be generally applicable. Different medical centers have different methods of varicose vein surgery, which can lead to different clinical outcomes. Hence, prospective multicenter research should be further carried out in the future.

## Conclusions

In short, we systematically compared the safety and efficacy of DS for VV patients with SVT below knee and patients had VV only. DS has been proven to be a safe and effective treatment for both patients with and without SVT below knee. and its short-term and long-term effects have been confirmed in our study. Taken together, our findings suggest that preoperative anticoagulation is not necessarily required. For VV patients with SVT below the knee, DS treatment can effectively reduce hospitalization time and lower in-patient expenses.

### Supplementary Information

**Additional file 1.**

## Data Availability

The data set analyzed by our research institute can be obtained from the corresponding author, if the reason is appropriate.

## Abbreviations

VV: Varicose veins
IS: Inpatient surgery
DS: Day surgery
SVT: Superficial venous thrombosis
PSM: Propensity score matching model
VTE: Venous thromboembolism
DVT: Deep vein thrombosis
VCSS: Venous clinical severity score
