# Supplementary material for: Comparison of day surgery between varicose veins with and without superficial venous thrombosis below knee: a propensity score-matched analysis
Source: BMC Cardiovasc Disord. 2023 Aug 3;23:387. doi: 10.1186/s12872-023-03398-2 (PMC10401813; doi:10.1186/s12872-023-03398-2)
Supplement: Supplementary file 1 — Additional file 1. [file 12872_2023_3398_MOESM1_ESM.pdf]

STROBE Statement—Checklist of items that should be included in reports of ***cohort studies***

	Item No	Recommendation	NO.	Relevant text for manuscript
**Title and abstract**	1	(a) Indicate the study's design with a commonly used term in the title or the abstract	3	This is a single-center retrospective study.
		(b) Provide in the abstract an informative and balanced summary of what was done and what was found	3	This study aimed to investigate whether the existence of SVT below knee affect the safety and efficacy of DS for VV patients.<br>Our results indicate DS is safe and effective for patients with VV, whether accompanied by SVT below the knee.
Introduction				
Background/rationale	2	Explain the scientific background and rationale for the investigation being reported	5-6	Moreover, advancements in endovenous technology have made it possible for clinicians to effectively treat VV through DS.<br>Superficial venous thrombosis (SVT) is a frequent complication of VV. It manifests as pain, erythema, and typically a palpable nodular mass. Although SVT can occur independently, VV remains the primary risk factor.<br>SVT patients typically require a period of standardized anticoagulant therapy, while DS is currently considered the mainstream treatment for VV.<br>It remains unclear the impact of SVT on the efficacy of DS for varicose veins patients.
Objectives	3	State specific objectives, including any prespecified hypotheses	6	To comprehensively evaluate the feasibility and efficacy of DS for VV patients complicated with SVT, we conducted this study and systematically compared it to patients with VV only.
Methods				
Study design	4	Present key elements of study design early in the paper	7	This is a single center retrospective cohort study
Setting	5	Describe the setting, locations, and relevant dates, including periods of recruitment, exposure, follow-up, and data collection	7	This is a single center retrospective cohort study, which retrospectively analyzed the clinical data of 593 VV patients treated in Sun Yat-sen Memorial Hospital of Sun Yat-sen University from 2015 to 2021.
Participants	6	(a) Give the eligibility criteria, and the sources and methods of selection of participants. Describe methods of follow-up	8-11	Eligibility criteria: VV patients with the CEAP grade of C2-C5 and at the age 18 to 75; SVT limited to calf.<br>Sources: patients treated in Sun Yat-sen Memorial Hospital of Sun Yat-sen

				University from 2015 to 2021.<br>Methods of follow-up:<br>Follow-up assessments were conducted at the third, sixth, and twelfth month after operation, during which the VCSS socre was obtained to evaluate the postoperative condition of the patients.<br>For those patients who were not able to return for follow-up, such as those residing too far away from our institution, we conducted phone calls with well-trained physicians to collect the necessary information.
		(b) For matched studies, give matching criteria and number of exposed and unexposed	7	To reduce the possible selection bias, a propensity score matching model (PSM) was used to balance the baseline data of the two groups of patients [19].<br>Patients complicated with SVT (59) were matched at a ratio of 1:2 with patients suffered VV only (118) finally.
Variables	7	Clearly define all outcomes, exposures, predictors, potential confounders, and effect modifiers. Give diagnostic criteria, if applicable	10	Perioperative clinical outcomes included operation time, number of incisions and perioperative adverse events. Perioperative adverse events included postoperative DVT, technical failure, skin burns, saphenous nerve injury, subcutaneous induration, and bleeding requiring intervention. Technical failure is defined as failure of venipuncture or failure to complete the operation after venipuncture.<br>Follow-up outcomes included VV recurrence, SVT formation, and DVT events. Also, we executed VCSS scores on patients at the third, sixth and twelfth month after operation. VCSS score was calculated in the term of 10 items. The higher the score is, the worse the lower limb veins are.
Data sources/ measurement	8*	For each variable of interest, give sources of data and details of methods of assessment (measurement). Describe comparability of assessment methods if there is more than one group	7-11	By consulting the electronic medical record, the demographic and clinical information such as patient gender, age, weight and basic diseases were extracted.<br>Doppler ultrasound was used to objectively evaluate the follow-up outcomes, included VV recurrence, SVT formation, and DVT events.<br>For those patients who were not able to return for follow-up, such as those residing too far away from our institution, we conducted phone calls with well-trained physicians to collect the necessary information.
Bias	9	Describe any efforts to address potential sources of bias	7	To reduce the possible selection bias, a propensity score matching model (PSM) was used to balance the baseline data of the two groups of patients.

Study size	10	Explain how the study size was arrived at	7	This is a single center retrospective cohort study, which retrospectively analyzed the clinical data of 593 VV patients treated in Sun Yat-sen Memorial Hospital of Sun Yat-sen University from 2015 to 2021.
Quantitative variables	11	Explain how quantitative variables were handled in the analyses. If applicable, describe which groupings were chosen and why	11	χ2 test or Fisher's exact test were used to compare categorical variables
Statistical methods	12	(a) Describe all statistical methods, including those used to control for confounding	10-11	To minimize selection bias and ensure comparability of baseline data between the two groups, we performed PSM. The propensity scores of each patient were calculated using a logistic regression model, and a 1:2 matching ratio was applied to match the propensity scores of the two groups. The value of caliper was 0.1. The baseline variables matched in our model included gender, age, weight, combined hypertension, CEAP grade and preoperative VCSS score. χ2 test or Fisher's exact test were used to compare categorical variables, student's t test or Wilcoxon rank sum test were used to compare continuous variables. Binary outcomes were calculated by logistic regression. All statistical tests were two tailed, P<0.05 was considered statistically significant. Our statistical analyses were carried out by the using of STATA 15 (STATACrop). .
		(b) Describe any methods used to examine subgroups and interactions		No subgroup analysis was set up in this study
		(c) Explain how missing data were addressed		This study was a single-center retrospective study. To ensure the quality of the study, patients with missing data were not included
		(d) If applicable, explain how loss to follow-up was addressed		None
		(e) Describe any sensitivity analyses		None
Results				
Participants	13*	(a) Report numbers of individuals at each stage of study—eg numbers potentially eligible, examined for eligibility, confirmed eligible, included in the study, completing follow-up, and analysed	12	593 patients diagnosed with VV were treated by DS, among which 59 patients had the additional complication of SVT. Raw data were analyzed by the using of PSM with a caliper set to 0.1. Matching results are summarized in Figure 1. Patients complicated with SVT (59) were matched at a ratio of 1:2 with patients

				who had VV only (118).
		(b) Give reasons for non-participation at each stage	12	Patients complicated with SVT (59) were matched at a ratio of 1:2 with patients who had VV only (118)
		(c) Consider use of a flow diagram		None
Descriptive data	14*	(a) Give characteristics of study participants (eg demographic, clinical, social) and information on exposures and potential confounders	12	The baseline characteristics of participant patients before and after PSM were depicted in Table 1.
		(b) Indicate number of participants with missing data for each variable of interest		This study was a single-center retrospective study. To ensure the quality of the study, patients with missing data were not included
		(c) Summarise follow-up time (eg, average and total amount)	10	Follow-up assessments were conducted at the third, sixth, and twelfth month after operation, during which the VCSS socre was obtained to evaluate the postoperative condition of the patients<br>We carried the last follow-up in December 2021.
Outcome data	15*	Report numbers of outcome events or summary measures over time	12-13	Among patients with VV only, perioperative adverse events consisted of 2 cases (1.69%) of burns, 2 cases (1.69%) of saphenous nerve injury, 46 cases (38.98%) of subcutaneous induration, and 1 case (0.85%) of deep vein thrombosis (DVT) occurrence. And among patients complicated with SVT, perioperative adverse events included 2 (3.39%) of saphenous nerve injury, 25 (42.37%) of subcutaneous induration and 1 (1.69%) of DVT occurrence.<br>AS showed in Table 3, VV recurrence occurred in 17 patients in group suffered VV only and 7 patients in SVT group during follow-up (14.41% VS 11.86%, P=0.641). No difference was found on the rate of SVT formation between SVT and non-SVT groups (5.08% VS 3.39%, P=0.892). Importantly, no cases of deep vein thrombosis (DVT) were reported during the follow-up period.
Main results	16	(a) Give unadjusted estimates and, if applicable, confounder-adjusted estimates and their precision (eg, 95% confidence interval). Make clear which confounders were adjusted for and why they were included	12-13	Based on the 12-month follow-up, the mean VCSS score for patients with VV only were 4.79, 3.36, and 2.74 at the 3rd, 6th, and 12th month, respectively, which were almost same as patients complicated with SVT (4.61, 3.52 and 2.63), as showed in Figure 2.<br>The operation time in the SVT group was statistically paralleled to that of the non-SVT group, with no significant difference observed (median=41 [39, 45] VS median=41 [38 ,44], P=0.726). Whereas, patients complicated with SVT

				had more incisions compared to those with VV only (median=6 [5, 7] VS median=4 [4, 5], $P < 0.001$).
		(b) Report category boundaries when continuous variables were categorized		None
		(c) If relevant, consider translating estimates of relative risk into absolute risk for a meaningful time period		None
Other analyses	17	Report other analyses done—eg analyses of subgroups and interactions, and sensitivity analyses		None
Discussion				
Key results	18	Summarise key results with reference to study objectives	15	Therefore, the results of our study provide strong evidence regarding the safety and efficacy of DS for VV patients with SVT.
Limitations	19	Discuss limitations of the study, taking into account sources of potential bias or imprecision. Discuss both direction and magnitude of any potential bias	18	Several limitations in the study should be addressed. First, being a retrospective study, we cannot eliminate all possible biases through PSM. For instance, the majority of patients included in our study had CEAP grades ranging from C2 to C5, while the more serious patients with active ulcer have not been adequately analyzed. Therefore, further research is required to investigate the safety and efficacy of DS for those patients with active ulcer (C6). Second, considering particles of SVT of thigh can be propagated into the deep venous system in endovenous ablation, only patients with SVT below knee were included in our study. Third, not all patients underwent lower extremity ultrasonography during follow-up, which means that there is a possibility that some cases of asymptomatic isolated DVT were not documented. At last, our study is a single center study, and the clinical outcomes observed may not be generally applicable. Different medical centers have different methods of varicose vein surgery, which can lead to different clinical outcomes. Hence, prospective multicenter research should be further carried out in the future.
Interpretation	20	Give a cautious overall interpretation of results considering objectives, limitations, multiplicity of analyses, results from similar studies, and other	15-17	PSM was performed from a large volume of data preexisted to balance the baseline characteristics between SVT and non-SVT group. As a retrospective cohort study, the raw baseline data of the two groups were unevenly distributed

relevant evidence

in age, VCSS score and CEAP grade. Previous studies have indicated that SVT is more prevalent among the elderly population. And worse VCSS score, as well as higher CEAP grade in SVT group may be attributed to the aggravation of venous symptoms by SVT and the residual pigmentation of local skin after the acute phase[10, 26].

Both SVT and non-SVT patients demonstrated similar clinical characteristics and distribution after matching, which suggested less likelihood of selection bias in studying the outcomes. In our propensity score matched cohort, patients in SVT group had higher number of surgical incisions compared to patients in non-SVT group. This can be attributed to surgeons tend to make an incision on the surface of SVT in shank for embolectomy for SVT cases. Though embolectomy was performed during surgery, it will not extend operation time, making operation time of two groups comparable. Most incisions were located in shallow skin and less than 2cm, and these incisions generally healed quickly and resulted in a short recovery time.

Perioperative outcomes indicate the safety and efficacy of DS for VV patients with SVT. The perioperative outcomes were acceptable in both SVT and non-SVT patients. No significant difference in the incidence of technical failure was showed in our research, as well as major perioperative adverse events. The occurrence of major adverse events such as skin burns, saphenous nerve injury, and postoperative deep vein thrombosis (DVT) was low and could be effectively managed with conservative therapy. Additionally, no bleeding requiring intervention occurred, which further suggested the safety of DS for both groups.

In our study, we focused on the high incidence of postoperative subcutaneous induration in both groups (38.98% in the non-SVT and 42.37% in the SVT). Induration along the trunk of GSV in this research, as in previous studies, has a high incidence rate in endovenous ablation, but mostly dissolved within 4 weeks (i.e. at the first review after discharge)[27].Traditionally, SVT was regarded as a disease with high risk of VTE and required standardized anticoagulant therapy for a period of time. However, our finding suggested that in VV patients with SVT, DS can be safely performed without the need for

				standardized anticoagulation before the operation.<br>Moreover, although no significant difference in incidence of postoperative DVT was find in our research between the two groups, two patients still experienced postoperative DVT. By reviewing the surgical records, we speculated that this could be attributed to the excessive use of foam sclerosing agents during the operation[28, 29]. Both patients suffered asymptomatic distal DVT patients, and the thrombus disappeared after a short period of after short-term therapeutic dose anticoagulation.<br>During the long-term follow-up, both groups showed a great decrease in VCSS score. VV clinical recurrence was observed in 7 (11.86%) patients with SVT and 17 (14.41%) patients without SVT, which suggested satisfactory long-term efficacy. SVT recurrence occurred in 3 (5.08%) patients with SVT and 4 (3.39%) patients without SVT during follow-up, and all cases were cured after treated with topical Hirudoid. Importantly, no patients suffered DVT during follow-up which also demonstrated the long-term safety of DS in VV patients with SVT.
Generalisability	21	Discuss the generalisability (external validity) of the study results	17	At last, our study is a single center study, and the clinical outcomes observed may not be generally applicable. Different medical centers have different methods of varicose vein surgery, which can lead to different clinical outcomes. Hence, prospective multicenter research should be further carried out in the future.
Other information				
Funding	22	Give the source of funding and the role of the funders for the present study and, if applicable, for the original study on which the present article is based	19	The project (including data analysis and manuscript writing) is sponsored by National Natural Science Foundation of China (Project approval number is 81800420).

*Give information separately for exposed and unexposed groups.

Note: An Explanation and Elaboration article discusses each checklist item and gives methodological background and published examples of transparent reporting. The STROBE checklist is best used in conjunction with this article (freely available on the Web sites of PLoS Medicine at http://www.plosmedicine.org/, Annals of Internal Medicine at http://www.annals.org/, and Epidemiology at http://www.epidem.com/). Information on the STROBE Initiative is available at http://www.strobe-statement.org.
